# Family with sequence similarity 107: A family of stress responsive small proteins with diverse functions in cancer and the nervous system (Review)

**DOI:** 10.3892/br.2014.243

**Published:** 2014-02-27

**Authors:** HIDEO NAKAJIMA, KEITA KOIZUMI

**Affiliations:** 1 Department of Oncology, Ageo Central General Hospital, Ageo, Saitama 362-8588, Japan; 2 Center for AIDS Research, Kumamoto University, Kumamoto 860-0811, Japan; 3 Research Center for Child Mental Development, Kanazawa University, Kanazawa, Ishikawa 920-8640, Japan

**Keywords:** family with sequence similarity 107, stress response, heat shock protein

## Abstract

Under conditions of acute stress, rapid adaptation is crucial for maximizing biological survival. The responses to environmental stress are often complex, involving numerous genes and integrating events at the cellular and organismal levels. The heat shock proteins (HSPs) are a family of highly conserved proteins that play critical roles in maintaining cell homeostasis and protecting cells under chronic and acute stress conditions. The genes for these stress-responding proteins are widely distributed in organisms, tissues and cells. HSPs participate in a variety of physiological processes and are associated with various types of disease. In this review, we focused on family with sequence similarity 107 (FAM107), a novel unique protein family that exhibits functional similarity with HSPs during the cellular stress response. This review aimed to summarize the biological properties of FAM107 in cancer and the nervous system.

## Introduction

**Figure 1:**
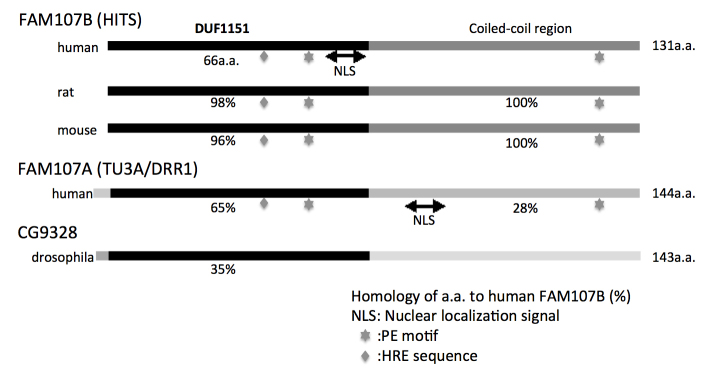
Comparison of the molecular structures of FAM107 proteins among different species. For rat and mouse FAM107B, human FAM107A and *Drosophila* CG9328, the homology of each protein to the human FAM107B protein sequence is expressed as a percentage (%). The NLS, PE and HRE motifs are indicated. FAM107, family with sequence similarity 107; HITS, heat shock-inducible tumor small protein; DUF1151, N-terminal domain of unknown function; NLS, nuclear localization signal; TU3A, Tohoku University cDNA clone A on chromosome 3; DRR1, downregulated in renal cell carcinoma gene 1; PE, proline-glutamic acid; HRE, histidine-arginine-glutamic acid sequence; a.a., amino acids.

Family with sequence similarity 107 (FAM107) members contain an N-terminal domain of unknown function (DUF1151) that is conserved across species, including mammalian, *Xenopus*, fish and *Drosophila*, with no homologous matches to other functionally conserved domains (Fig. 1). This family includes several hypothetical eukaryotic proteins with largely undetermined functions. Mammals have two genes, FAM107A and FAM107B, which encode for proteins of 144 and 131 amino acids (aa), respectively. The C-terminal variable regions of FAM107 members have a coiled-coil domain that has been identified in several nuclear proteins, including transcription factors, suggesting a role for FAM107 members in regulating gene transcription. Protein-protein interaction analyses demonstrated that FAM107A and FAM107B interact with transcriptional adaptor (Tada)2α and 3α, respectively (1–3). Tada2α and 3α are core proteins of the histone acetyltransferase (HAT) complex, suggesting that FAM107 proteins modulate the structure and function of HAT complexes, resulting in gene transcriptional modifications and protein acetylation (4,5).

Although the major physiological functions of FAM107 proteins remain to be investigated, the present review aimed to summarize the currently available biological information regarding the role of FAM107 members in cancer and neural cells.

## 2. FAM107 in cancer

FAM107A has been designated Tohoku University cDNA clone A on chromosome 3 (TU3A) and is also referred to as downregulated in renal cell carcinoma gene 1 (DRR1). FAM107A is a candidate tumor suppressor gene located on chromosome 3p21.1 (6,7). Several studies indicated that FAM107A expression is downregulated in various types of cancer, such as non-small-cell lung, renal cell and prostate cancers and astrocytoma by epigenetic silencing, including promoter hypermethylation (6–11). The forced expression of FAM107A was shown to suppress tumor cell proliferation and induce apoptosis (7,11–13). Thus, FAM107A was considered as a tumor suppressor gene due to its decreased expression in various types of cancer and since inducing FAM107A expression suppresses cancer cell proliferation and induces apoptosis. However, FAM107A was also found to be highly expressed in the invasive component of gliomas and may drive tumor invasion by modulating the cytoskeleton (14,15). Thus, the physiological roles and functions of FAM107A in cancer remain controversial.

Despite accumulating information regarding FAM107A, the available biological data on FAM107B are currently limited. In humans, the FAM107B protein is encoded by a gene on chromosome 10p13. This protein consists of 131 aa and its sequence is ~98% identical with mouse and rat homologues (Fig. 1). FAM107A and FAM107B proteins exhibit a 65% sequence similarity in their DUF1151 regions.

The most notable characteristic of FAM107B, unlike FAM107A, is that the FAM107B gene has a promoter region with heat shock transcription factor 1 (HSF1)-binding sites, and FAM107B transcription is increased following heat-shock or hyperthermia treatment. Thus, its protein was designated as heat shock-inducible tumor small protein (HITS) (16). Our preliminary investigation found that the level of HITS expression in gastrointestinal cancer cells was significantly lower compared to that in normal epithelial cells, although its expression pattern and intensity varied among cancers of different histological types. HITS expression was decreased during the process underlying colorectal adenoma-to-carcinoma transition. In addition, HITS expression was decreased in intestinal-type gastric adenocarcinomas, but not in diffuse-type or mucinous adenocarcinomas.

Multiple organ tissue microarray analyses revealed that HITS expression was decreased in other tumor tissues, such as breast, thyroid, testicular and uterine cervical in a histological type-specific manner (17). HITS expression intensity was found to be inversely correlated with primary tumor size [T-value in tumor-node-metastasis (TNM) grading] in breast and thyroid cancers, but not with lymph node metastasis (N-value). For breast cancers, the statistical correlation analysis for HITS expression and the clinicopathological parameters of human epidermal growth factor 2 (HER2), estrogen receptor, progesterone receptor (PR), Ki-67 and p53 revealed that HITS expression intensity was positively correlated with the expression of HER2 and Ki-67, but was inversely correlated with PR expression. Accordingly, HITS expression was markedly lost in HER2-negative, Ki-67-negative, PR-positive and desmoplastic reaction-negative type of breast cancer, which is considered to be a non-aggressive or indolent phenotype.

As regards uterine cervical diseases, HITS expression was significantly lost in invasive squamous cell carcinoma, but not in cervical intraepithelial neoplasia (CIN). Infection by human papilloma virus (HPV) is known to induce the development of cervical cancer, due to the strong causal association between HPV infection, CIN and invasive carcinoma (18). As certain CIN lesions may progress to invasive cancer over a period of 10–20 years, our findings suggest that HITS expression is lost during the progression of CIN to invasive carcinoma.

**Figure 2:**
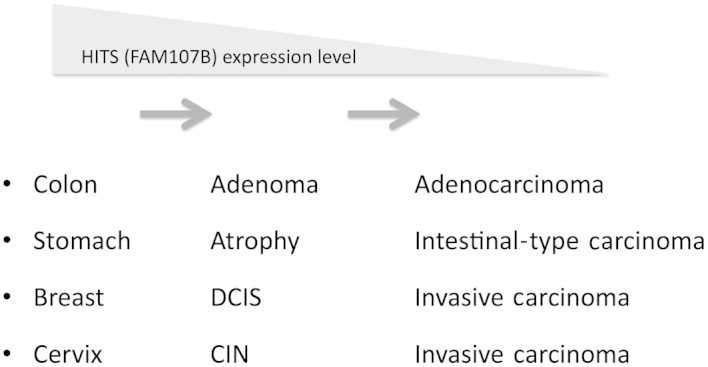
Conceptual loss of HITS expression during carcinogenesis. The HITS expression level gradually decreases during the process leading from a precancerous or borderline malignancy region to carcinoma in several organs. HITS, heat shock-inducible tumor small protein; FAM107, family with sequence similarity 107; DCIS, ductal carcinoma *in situ*; CIN, cervical intraepithelial neoplasia.

Considering that HITS expression was lost during the course of tumor progression in terms of TNM grading T-values, we hypothesize that HITS expression declines gradually during the prolonged transition from preneoplastic or early neoplastic lesions, such as ductal carcinoma *in situ* in the breast, intestinal metaplasia in the stomach, tubular adenoma in the colon and CIN in the uterine cervix, to invasive cancers (Fig. 2). By contrast, HITS expression is preserved in aggressive types of cancers, such as scirrhous-type gastric and breast cancers, which are characterized by distinct genetic alterations and rapid growth or invasion.

Furthermore, it was reported that a point mutation in the C-terminal region of HITS (chromosome 10: 14603968 C•G→T•A transition) was frequently observed in genomic analyses of basal-like breast cancer (19). The protein sequence of the C-terminal coiled-coil region is unique to HITS, which suggests its role in tumorigenicity through the transcriptional regulation of oncogenes or tumor suppressor genes. Therefore, it was hypothesized that, in an aggressive type of breast cancer, such as basal-like or scirrhous-type, HITS expression is relatively preserved, but its antioncogenic function is lost by this genetic mutation.

In addition, forced expression of HITS was shown to inhibit cancer cell proliferation in response to growth factors *in vitro* and tumor xenograft growth *in vivo* (16,17). HITS may be considered as a candidate tumor suppressor gene, since loss of HITS expression was commonly observed in cancers of various organs, resulting in tumor development and proliferation, similar to FAM107A. We hypothesize that HITS expression affects the growth of primary tumors during development, but does not affect invasion or metastasis, such as scirrhous-type tumor spread or lymph node metastasis. Consequently, HITS is a potential tumor suppressor protein with the unique characteristic of its transcription being induced by heat shock stimulation. This is a particularly distinct characteristic of HITS, as other heat shock proteins and HSF1 are considered to exhibit certain oncogenic activities (20,21).

## 3. FAM107 in neuron

**Figure 3:**
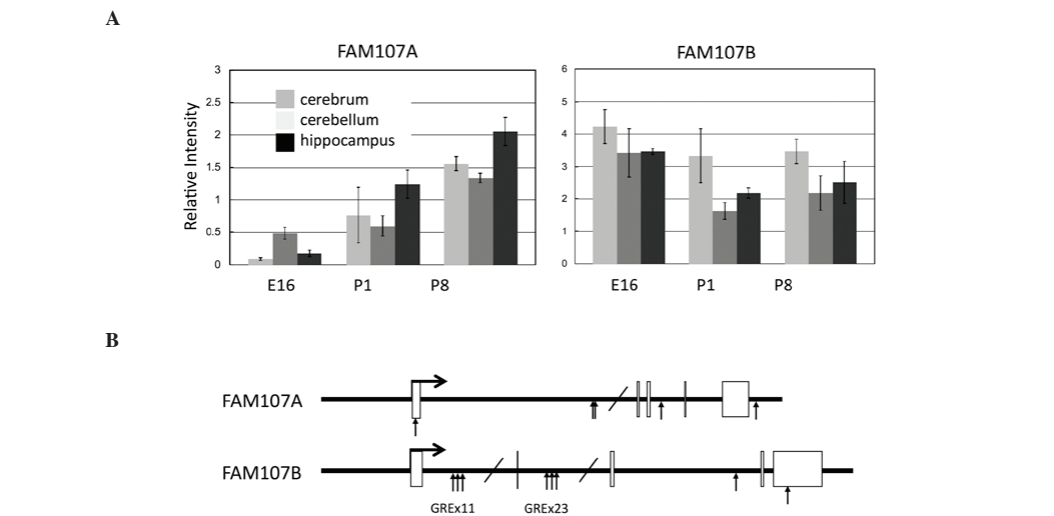
Control of FAM107 protein expression in neural cells. (A) Developmental expression profiles of FAM107A and FAM107B in the brain determined by semi-quantitative RT-PCR. (B) Putative glucocorticoid responsive elements (GRE) located in FAM107A and FAM107B genomic regions (arrows) (36,37). FAM107, family with sequence similarity 107; RT-PCR, reverse transcription-polymerase chain reaction.

FAM107A and FAM107B are expressed in normal brain tissues (Fig. 3A) and FAM107A (DRR1) was shown to play critical roles in neural cell survival, migration and spine formation (14,22,23). Considering the molecular similarities between FAM107A and FAM107B, FAM107B (HITS) may also play critical roles during brain development.

FAM107A is widely expressed in various normal tissues and its expression is particularly high in the brain (7,13,14,22). In primary cultures of rat fetal cerebral cortex, FAM107A was found to be differentially expressed in neurons and enriched in axon projections and cell nuclei (14,22). A study using RNA interference knockdown indicated that FAM107A is essential for neural cell survival (22).

Studies on glioma cells may help elucidate the functions of FAM107A in neural cells. Le *et al* (14) indicated that FAM107A expression co-localized with F-actin stress fiber focal adhesions (FAs) and membrane ruffles in U251 glioma cells that were transfected with FAM107A. The proline-glutamic acid (PE) motif (65–66/122–123 aa in human FAM107A) is critical for the association of FAM107A with F-actin and its intercellular localization (Fig. 1). The PE motif also binds to a light chain subunit (LC2) of microtubule-associated protein 1 to modulate microtubule assembly, which requires the histidine-arginine-glutamic acid (HRE) sequence motif located in the N-terminal region aa of FAM107A. In fact, these 2 motifs are also conserved in human, mouse and rat FAM107B (Fig. 1). FAM107A overexpression was shown to facilitate U251 cell invasion, whereas a PE or HRE motif mutation suppressed the effect of overexpression. It is hypothesized that FAM107A acts as an actin-microtubule cross-linker to organize the cytoskeletons essential for FAs modulation and glioma invasion. Similarly, FAM107A may regulate neural cell migration in the developing brain. As regards modulation of the cytoskeleton, Akt signaling may play a crucial role. FAM107A overexpression was shown to increase Akt phosphorylation at the Thr308 and Ser473 sites, which recruit Akt localization to FAs, without affecting total Akt expression (15).

Schmidt *et al* (23) reported that FAM107A is a stress response protein in the brain. The stress associated with maternal separation (neonatal) or food deprivation (adult) significantly increased FAM107A mRNA expression in the paraventricular nucleus and CA3 regions of the hippocampus. Treating adult mice with dexamethasone (DEX; a glucocorticoid agonist) also resulted in increased FAM107A mRNA expression in the same brain regions. Increased FAM107A expression induced by DEX was also reported in cultured astrocytes (24). FAM107A transcription may be directly regulated by glucocorticoid receptors (5). In fact, a number of glucocorticoid responsive elements are found in FAM107A genomic regions, which is also the case for the FAM107B gene (Fig. 3B).

In the hippocampus, FAM107A expression was found to be preferentially localized in the presynaptic regions of neuron synapses and its overexpression reduced spine density. FAM107A overexpression also caused a reduction in the magnitude of long-term potentiation that improves cognitive behavior (23).

FAM107A may be associated with certain types of human psychiatric disorders. FAM107A mRNA expression was increased in postmortem RNA samples from the dorsolateral prefrontal cortex (Brodman area 46) of patients suffering from schizophrenia and bipolar disorder (25). A number of studies suggested that human chromosome 3p14, the location of FAM107A, is linked to psychiatric disorders, such as schizophrenia (26–28), bipolar disorder (25,29–32) and Asperger syndrome (33–35). Although there is currently no direct evidence, FAM107A may be a candidate gene associated with these psychiatric disorders.

## 4. Conclusion

The expression of FAM107A and FAM107B proteins is prominent in neural cells, whereas their expression is downregulated in cancer cells. The two proteins appear to affect cytoskeleton rearrangements and are involved in cell migration and expansion. The molecular mechanisms underlying the diverse biological functions of FAM107 remain unclear. In particular, the functions and the molecular interactions of the N-terminal conserved domain (DUF1151) of FAM107 requires further investigation as they may play crucial roles in the interactions with other proteins for transducing cell signals and modulating gene transcription, which may be common among FAM107 proteins. Further investigations are required to provide evidence for the biological importance of this well-conserved protein family in cancer and the nervous system, which may lead to improved clinical diagnosis and the development of therapeutic uses for FAM107 proteins.

## References

[1] Rual JF, Venkatesan K, Hao T (2005). Towards a proteome-scale map of the human protein-protein interaction network. Nature.

[2] Ewing RM, Chu P, Elisma F (2007). Large-scale mapping of human protein-protein interactions by mass spectrometry. Mol Syst Biol.

[3] Stelzl U, Worm U, Lalowski M (2005). A human protein-protein interaction network: a resource for annotating the proteome. Cell.

[4] Wang YL, Faiola F, Xu M, Pan S, Martinez E (2008). Human ATAC is a GCN5/PCAF-containing acetylase complex with a novel NC2-like histone fold module that interacts with the TATA-binding protein. J Biol Chem.

[5] Frijters R, Fleuren W, Toonen EJ (2010). Prednisolone-induced differential gene expression in mouse liver carrying wild type or a dimerization-defective glucocorticoid receptor. BMC Genomics.

[6] Yamato T, Orikasa K, Fukushige S, Orikasa S, Horii A (1999). Isolation and characterization of the novel gene, TU3A, in a commonly deleted region on 3p14.3→p14.2 in renal cell carcinoma. Cytogenet Cell Genet.

[7] Wang L, Darling J, Zhang JS, Liu W, Qian J, Bostwick D, Hartmann L, Jenkins R, Bardenhauer W, Schutte J, Opalka B, Smith DI (2000). Loss of expression of the DRR 1 gene at chromosomal segment 3p21.1 in renal cell carcinoma. Genes Chromosomes Cancer.

[8] van den Boom J, Wolter M, Blaschke B, Knobbe CB, Reifenberger G (2006). Identification of novel genes associated with astrocytoma progression using suppression subtractive hybridization and real-time reverse transcription-polymerase chain reaction. Int J Cancer.

[9] Vanaja DK, Ballman KV, Morlan BW (2006). PDLIM4 repression by hypermethylation as a potential biomarker for prostate cancer. Clin Cancer Res.

[10] Awakura Y, Nakamura E, Ito N, Kamoto T, Ogawa O (2008). Methylation-associated silencing of TU3A in human cancers. Int J Oncol.

[11] Liu Q, Zhao XY, Bai RZ (2009). Induction of tumor inhibition and apoptosis by a candidate tumor suppressor gene DRR1 on 3p21.1. Oncol Rep.

[12] Kholodnyuk ID, Kozireva S, Kost-Alimova M, Kashuba V, Klein G, Imreh S (2006). Down regulation of 3p genes, LTF, SLC38A3 and DRR1, upon growth of human chromosome 3-mouse fibrosarcoma hybrids in severe combined immunodeficiency mice. Int J Cancer.

[13] Zhao XY, Liang SF, Yao SH (2007). Identification and preliminary function study of *Xenopus laevis* DRR1 gene. Biochem Biophys Res Commun.

[14] Le PU, Angers-Loustau A, de Oliveira RM (2010). DRR drives brain cancer invasion by regulating cytoskeletal-focal adhesion dynamics. Oncogene.

[15] Dudley A, Sater M, Le PU (2013). DRR regulates AKT activation to drive brain cancer invasion. Oncogene.

[16] Nakajima H, Ishigaki Y, Xia QS (2010). Induction of HITS, a newly identified family with sequence similarity 107 protein (FAM107B), in cancer cells by heat shock stimulation. Int J Oncol.

[17] Nakajima H, Koizumi K, Tanaka T (2012). Loss of HITS (FAM107B) expression in cancers of multiple organs: Tissue microarray analysis. Int J Oncol.

[18] Bosch FX, de Sanjosé S (2003). Chapter 1: Human papillomavirus and cervical cancer - burden and assessment of causality. J Natl Cancer Inst Monogr.

[19] Ding L, Ellis MJ, Li S (2010). Genome remodelling in a basal-like breast cancer metastasis and xenograft. Nature.

[20] Dai C, Whitesell L, Rogers AB, Lindquist S (2007). Heat shock factor 1 is a powerful multifaceted modifier of carcinogenesis. Cell.

[21] Khalil AA, Kabapy NF, Deraz SF, Smith C (2011). Heat shock proteins in oncology: diagnostic biomarkers or therapeutic targets?. Biochim Biophys Acta.

[22] Asano Y, Kishida S, Mu P, Sakamoto K, Murohara T, Kadomatsu K (2010). DRR1 is expressed in the developing nervous system and downregulated during neuroblastoma carcinogenesis. Biochem Biophys Res Commun.

[23] Schmidt MV, Schulke JP, Liebl C (2011). Tumor suppressor down-regulated in renal cell carcinoma 1 (DRR1) is a stress-induced actin bundling factor that modulates synaptic efficacy and cognition. Proc Natl Acad Sci USA.

[24] Slezak M, Korostynski M, Gieryk A (2013). Astrocytes are a neural target of morphine action via glucocorticoid receptor-dependent signaling. Glia.

[25] Shao L, Vawter MP (2008). Shared gene expression alterations in schizophrenia and bipolar disorder. Biol Psychiatry.

[26] Tastemir D, Demirhan O, Sertdemir Y (2006). Chromosomal fragile site expression in Turkish psychiatric patients. Psychiatry Res.

[27] Paunio T, Arajarvi R, Terwilliger JD (2009). Linkage analysis of schizophrenia controlling for population substructure. Am J Med Genet B Neuropsychiatr Genet.

[28] Greenwood TA, Swerdlow NR, Gur RE (2013). Genome-wide linkage analyses of 12 endophenotypes for schizophrenia from the Consortium on the Genetics of Schizophrenia. Am J Psychiatry.

[29] Cichon S, Schumacher J, Muller DJ (2001). A genome screen for genes predisposing to bipolar affective disorder detects a new susceptibility locus on 8q. Hum Mol Genet.

[30] Marcheco-Teruel B, Flint TJ, Wikman FP (2006). A genome-wide linkage search for bipolar disorder susceptibility loci in a large and complex pedigree from the eastern part of Cuba. Am J Med Genet B Neuropsychiatr Genet.

[31] Etain B, Mathieu F, Rietschel M (2006). Genome-wide scan for genes involved in bipolar affective disorder in 70 European families ascertained through a bipolar type I early-onset proband: supportive evidence for linkage at 3p14. Mol Psychiatry.

[32] Mathieu F, Dizier MH, Etain B (2010). European collaborative study of early-onset bipolar disorder: evidence for genetic heterogeneity on 2q14 according to age at onset. Am J Med Genet B Neuropsychiatr Genet.

[33] Ylisaukko-oja T, Nieminen-von Wendt T, Kempas E (2004). Genome-wide scan for loci of Asperger syndrome. Mol Psychiatry.

[34] Rehnstrom K, Ylisaukko-oja T, Nieminen-von Wendt T (2006). Independent replication and initial fine mapping of 3p21–24 in Asperger syndrome. J Med Genet.

[35] Salyakina D, Ma DQ, Jaworski JM (2010). Variants in several genomic regions associated with asperger disorder. Autism Autism Res.

[36] So AY, Chaivorapol C, Bolton EC, Li H, Yamamoto KR (2007). Determinants of cell- and gene-specific transcriptional regulation by the glucocorticoid receptor. PLoS Genet.

[37] Kuo T, Lew MJ, Mayba O, Harris CA, Speed TP, Wang JC (2012). Genome-wide analysis of glucocorticoid receptor-binding sites in myotubes identifies gene networks modulating insulin signaling. Proc Natl Acad Sci USA.

